# Targeting in Language: Unifying Deixis and Anaphora

**DOI:** 10.3389/fpsyg.2020.02016

**Published:** 2020-09-01

**Authors:** Leonard Talmy

**Affiliations:** Department of Linguistics and Center for Cognitive Science, University at Buffalo, The State University of New York, Buffalo, NY, United States

**Keywords:** deixis, anaphora, targeting, cues, trigger, demonstratives, pronouns, joint attention

## Abstract

This article proposes that a single cognitive system underlies the two domains of linguistic reference traditionally termed anaphora and deixis. In anaphora, the referent is an element of the current discourse itself, whereas in deixis, the referent is outside the discourse in its spatiotemporal surroundings. This difference between the lexical and the physical has traditionally led to distinct theoretical treatments of such referents. We propose instead that language engages a single linguistic/cognitive system–“targeting”–to single out a referent, whether it is speech-internal or speech-external. To outline this system: As a speaker communicates with a hearer, her attention can come to be on something in the environment–her “target”–that she wants to refer to at a certain point in her discourse. This target can be located near or far in either the speech-external (deictic) or the speech-internal (anaphoric) environment. She thus needs the hearer to know what her intended target is and to have his attention on it jointly with her own at the relevant point in her discourse. The problem, though, is how to bring this about. Language solves this problem through targeting. First, at the intended point in her discourse, the speaker places a “trigger”–one out of a specialized set of mostly closed-class forms. English triggers include *this/these*, *that/those*, *here*, *there*, *yonder*, *now*, *then*, *therefore*, *thus*, *so*, *such*, *yay*, *the*, personal pronouns, relative pronouns, and tense markers. Next, on hearing the trigger, the hearer undertakes a particular three-stage procedure. In the first stage, he seeks all available “cues” to the target. Such cues belong to 10 distinct categories, representing 10 different sources of information about the target. In the second stage, he combines these cues so as to narrow down to the one intended target and rule out alternative candidates. In the third stage, he maps the concept of the target he has found back onto the original trigger for integration with the sentence’s overall reference. This article is based on the overview portion of a book–*The Targeting System of Language*, MIT Press, 2018.

## Introduction

This study proposes that a single linguistic/cognitive system underlies two domains of linguistic reference, those traditionally termed anaphora and deixis. Broadly, an anaphoric referent is an element of the current discourse, whereas a deictic referent is outside the discourse in the spatiotemporal surroundings^1^. This is a distinction made between the lexical and the physical, one that has traditionally led to distinct theoretical treatments of the corresponding referents. Our proposal, on the contrary, is that language engages the same cognitive system to single out a referent whether it is speech-internal or speech-external. This single system, here named “targeting,” can be outlined as follows.

As a speaker communicates with a hearer, her attention can come to be on something in the environment that she wants to refer to at a certain point in her discourse. This object of her attention will be called her *target*. Such a target can be located near or far in either the *speech-internal* or the *speech-external* environment–that is, in traditional terms, can be either anaphoric or deictic. To communicate about such a target, she needs the hearer to know what it is and to have his attention on it jointly with her own at the relevant point in her discourse. The problem, though, is how to bring this about. She cannot somehow directly reach into the hearer’s cognition, take hold of his attention, and place it on her selected target at the intended moment.

A particular language-mediated process solves this problem. In this process, the speaker places a specialized lexical form at the relevant point in her discourse and, on hearing this, the hearer undertakes a specialized procedure. Her form is here called a *trigger* because it initiates, or “triggers,” his procedure. Every language has a particular set of mostly closed-class triggers. The English set includes *this*/*these*, *that*/*those*, *here*, *there*, *yonder*, *now*, *then*, *therefore*, *thus*, *so*, *such*, *yay*, *the*, personal pronouns, relative pronouns, and tense markers. Such triggers are not simply static “placeholders,” as some linguistic approaches view them, but in effect actively direct the hearer to undertake his procedure.

For its part, in turn, the hearer’s procedure has three stages. In the first stage, the trigger directs the hearer to find certain elements of information to which he does have ready access. These elements of information function as *cues* to the speaker’s intended target. Such cues have so far been found to belong to 10 distinct categories, representing 10 different sources of information. This first stage can thus be called the *trigger-to-cues* stage.

In the second stage, equipped with the cues that he has ascertained, the hearer uses them in combination to determine the speaker’s intended target. The cues together thus guide him toward the target, which he could not have known directly. Generally, each cue rules in some candidates for target status while ruling out others. In association, the cues thus enable the hearer to narrow down to the target, singling it out from alternative candidates. This second stage of the procedure can accordingly be called the *cues-to-target* stage.

In the third stage, having determined the target, the hearer maps his concept of it back onto the trigger in the speaker’s sentence. He relates this concept to the full conceptual content of the sentence in accord with the trigger’s syntactic relation to the sentence. He thus has his attention on the target jointly with that of the speaker at the point of the discourse, and with the relationship to it, that she had intended. This stage can then be called the *target-back-to-trigger* stage.

This whole interaction rests on a coordination of the speaker’s and the hearer’s cognitive processing. As part of her cognitive processing, the speaker aims to get the hearer’s attention jointly with her own on her target at a particular point in her discourse, selects the appropriate trigger to insert at that point, and ensures that cues in sufficient quantity and informativeness are available for the hearer to use to determine that target^2^. In turn, as part of his cognitive processing, the hearer perceives the trigger and, in consequence, carries out the three-stage procedure in which he finds the cues, determines the target, and integrates the concept of it back into the discourse, there to join his attention on it with that of the speaker.

This entire sequence–including the selection of a trigger, the three stages with their use of cues, and the cognitive processing of both speaker and hearer throughout–will be called *targeting*. Such targeting is understood as a linguistic/cognitive system that equally underlies both anaphora and deixis, in which they are unified as an essentially single phenomenon.

This targeting system is, then, the central topic of the present study. Its distinguishing features can be summarized as follows. Deixis and anaphora both rest on a trigger-initiated three-stage procedure–engaged in by a speaker and a hearer–in which the hearer finds cues, uses them to determine the speaker’s intended target, and maps the concept of that target back onto the trigger and into its sentence. The cues to the target fall into 10 categories representing 10 different sources of information. This “targeting” process is a single linguistic and cognitive system in which deixis and anaphora are unified. The cognitive processing of both speaker and hearer in this targeting system can, in many respects, be inferred and built into the analysis.

The present analysis can be distinguished from others, first, with regard to the relation between anaphora and deixis. As Consten (2003) suggests, most approaches highlight the differences between the two domains or simply focus on one of them. For example, Mitkov (1999) and Ariel (2014) focus on anaphora, while Diessel (1999, 2013), Levinson (2003), and Chilton (2014) focus on deixis.

To be sure, a few treatments have also highlighted the similarities or the commonalities between the domains. Authors with this approach include Bühler (1934), Peirce (1955) within semiotics, Silverstein (1976) and Hanks (2011) in their treatment of indexicality within linguistic anthropology, Consten himself (who sees a fuzzy boundary, parallelism, and coordination between the two domains), and Recanati (2005) within the tradition of language philosophy. Even Halliday and Hasan’s (1976) labeling of anaphora and deixis, respectively, as “endophora” and “exophora”–with prefixes referring to “inside” and “outside”–suggests an awareness of the two domains’ relatedness. However, this minority group has no counterpart of the explanatory system for unifying the two domains proposed in the targeting account.

Furthermore, the three-stage targeting procedure proposed here seemingly has no counterpart in other approaches. Specifically, no counterpart exists for the first stage, where a trigger directs a hearer to search for cues to a target–cues in 10 categories with distinct information sources. None exists for our second stage where such cues are all combined by the hearer in accord with certain governing principles to zero in on the target, and none exists for our third stage, where the hearer maps the concept of the target back onto the trigger for integration into its sentence.

The present article is based on the introductory sections of a book–*The Targeting System of Language* (Talmy, 2018; the MIT Press has kindly permitted this adaptation). Those sections serve there as an overview of an extensively laid-out framework. Standing alone, this article is also intended as an overview to the full framework. As an overview, it necessarily omits mention of numerous immediately relevant issues, but many of these are analyzed in detail in the book. Both the article and the book are set within the theoretical framework of “cognitive semantics” as put forth in Talmy (2000a,b, 2011).

As in traditional linguistics, the examples presented below and in the book as a whole are not observed but constructed, an option based on the following consideration. In science generally, two main methods might be cited for engaging with an area under examination, both of them valuable. In one, the researcher adopts the perspective of ecological validity, observing the naturally occurring patterns of interacting elements of the area within a context extended in both space and time. In the other method, the researcher controls the elements of the area and systematically manipulates them–for instance, holding other elements constant while varying one so as to investigate it in isolation. This method can reveal certain deep features of the area’s organization and operation not readily accessible otherwise. It is this second method that is realized in our technique of constructing examples.

The second method is further realized by psycholinguistic experimentation. The beginnings of such experimentation have been reported, e.g., on deixis by Bangerter (2004); Coventry et al. (2008, 2014); Imai (2009), and Peeters et al. (2015) and on anaphora by McKoon and Ratcliff (1980) and Sanford and Garrod (1989). The book, with its detailed theoretical framework, serves as a call for much further experimentation.

## Survey of the Ten Cue Categories

As mentioned, cues to a target can be analyzed as belonging to 10 distinct categories that represent 10 different sources of information about the target. These ten categories are outlined here. They can in turn be placed into five groups of two categories each. For simplicity in the survey, the categories are illustrated only with speech-external targets, but speech-internal targets are treated in section “Interaction of Compatible Cues to a Speech-Internal Target” below and, extensively so, in the book that this article is based on.

Each example in this survey includes one or more cues additional to the cue being illustrated. In fact, two or more cues are always needed in any given case for a hearer to determine a speaker’s intended target. Such cues are usually in different categories but sometimes are in the same category.

Any two such cues will have one of three *concordance relations* to each other. In two of these relations, the cues are compatible. The cues then either *corroborate* each other, providing the same information about the target, or *complement* each other, providing different information about the target. In the present survey, the cues included in each example have one of these two compatible relations. In the third relation, two cues *conflict* with each other, providing incompatible information about the target, but such conflict typically initiates a constructive resolution in the hearer that again helps guide him to the target. Section “Interaction of Incompatible Cues to a Speech-External Target Broader Systems” illustrates this conflict relation.

### The Lexical Cue Categories

In one group of two categories–the *lexical cue categories*–the cues to the target are provided by lexical forms in the speaker’s utterance. In one category, the cues are provided by the trigger and, in the other, by forms around the trigger.

#### Core Cues

The trigger that a speaker includes in an utterance not only initiates the three-stage targeting procedure in the hearer but, in addition, is always lexicalized to provide cues to certain characteristics of the target. These are here called *core cues*. For example, a speaker, without using manual or ocular gestures, might say either (1a) or (1b) while opening the door to his lab to let a visitor peer inside, where a woman and several machines are located.

(1) a. She is new here.
b. These are new here.

If he says (1a), the trigger *she* provides the core cues that the target has the characteristics of being uniplex, an entity, animate, female, and third-person (i.e., not the speaker or the hearer). In surveying the lab, the hearer perceives that one part of its contents, the woman there, exhibits these five characteristics. These perceivable characteristics then function as targetive cues (see below). The hearer combines these two types of cues, the core cues and the targetive cues–which corroborate each other–and settles on the woman as the speaker’s probable intended target.

If the speaker instead says (1b), the trigger *these* provides the core cues that the target has the characteristics of being multiplex, entities, proximal^3^, third-person, and–in the present construction–inanimate. In surveying the lab, the hearer will now likely select the machines as the speaker’s intended target.

In the third stage of the targeting process for either sentence, the hearer, having determined the target, maps the concept of it back onto the trigger. In accord with the trigger’s syntactic relation to the sentence^4^, the hearer then integrates that concept into the overall conception expressed by the sentence. In the present cases, where the trigger is a subject nominal in construction with the predicate adjective *new*, the hearer ascribes the concept of “newness” to the concept of the target–that is, the woman or the machines. This third stage will not be described in the rest of the survey.

#### Co-form Cues

The linguistic constituents located around a trigger are here called its *co-forms*. A *co-form cue*, then, consists of any information provided by a co-form that helps the hearer determine the target of that trigger. The further a constituent is from a given trigger, the less likely it is to provide a co-form cue relevant to that trigger, and the less it would be regarded as a co-form of it.

To illustrate, suppose that a customer in a pet shop that has only one parrot among its animals goes up to the clerk behind the counter and, without gesturing manually or looking, says (2):

(2) That is the kind of parrot I like.

The trigger in the speaker’s utterance, *that*, directs the hearer, that is, the clerk, to look for cues and use them to find a target. It also provides the core cues that that target has the properties of being uniplex, an entity, distal, and third-person. However, the hearer cannot narrow down to the target with these core cues alone because too many components in the scene have these properties. In the same utterance, the co-form *parrot* provides the co-form cues that the target has the properties of being a single entity with the identity of a parrot. These core and co-form cues corroborate each other in one respect–in indicating that the target is a unitary entity. They also complement each other, with the core cue indicating that the target’s location is distal and the co-form cue indicating that its identity is that of a parrot. As hearer, the clerk will combine these cues to single out the one parrot in the shop as the speaker’s intended target.

### The Bodily Cue Categories

In another group of two categories–the *bodily cue categories*–the cues are provided by the body of one of the speech participants. Those of the gestural cue category consist of movements or configurations of parts of the speaker’s body that she produces volitionally, while those of the corporal cue category consist simply of the location of the speaker’s or the hearer’s whole body.

#### Gestural Cues

Apart from the use of the mouth for speaking, any movement and/or configuration that a speaker volitionally produces with her body to communicate to a hearer is here considered a “gesture.” A gesture that a speaker produces in association with a trigger specifically in order to provide a cue to a target is then called a *targeting gesture*. The cue that such a targeting gesture provides is a *gestural cue*.

To illustrate, suppose that a speaker says (3) to a guest standing beside her, while pointing toward one corner of a table across the room from them. That corner is clear in front, but a bottle of wine is standing about a foot back from its edge. (An exclamation point placed before a word here indicates heightened stress on that word).

(3) You can put your glass down right!-there.

The trigger *there* in the speaker’s utterance not only alerts the hearer to find a particular target but also provides the core cues that this target is distal and is a location, not an entity. However, this cue by itself is not enough, given the multitude of distal locations in the situation. The speaker’s gesture also provides a gestural cue to the target. By our analysis, this gesture leads the hearer to imagine an intangible line extending from the speaker’s finger to the table’s corner where the target is. Such an intangible line is one instance of an elaborate system of *fictive chains*^5^.

In combination, these core and gestural cues corroborate each other in indicating that the target is distal. They also complement each other in indicating, respectively, that the target is a location and that this location is situated where the imaginal line terminates at the table’s corner. Integrating these cues, the hearer will select the surface of the table at the corner as the intended target–singling it out from other regions of space in the room. He will not select the bottle as the target–though it is equally included by the pointing gesture–because it is an entity, not a location.

The hearer is additionally guided toward this intended target by co-form cues from the phrase *put your glass down* and by the epistemic cues that they evoke (see below), namely, the knowledge that a glass is normally placed by resting it on a clear horizontal surface. This knowledge corroboratively rules in the table’s surface and rules out the bottle as the target.

Suppose now that the speaker, while pointing as before, instead says (4):

(4) Could you please bring!-that over to me?

Here the trigger *that* provides the core cues that the target is uniplex, distal, third-person, and an entity, not a location. The gestural cue is the same as above. Combining these core and gestural cues, the hearer will now select the bottle of wine in the corner portion of the table as the intended target, singling it out from other entities in the room. He will not select the portion of the table’s surface included by the gesture because it is a location, not an entity. The hearer is now additionally guided by co-form cues from the expression *bring over* and the epistemic cues they evoke: the knowledge that a person can bring only something he can readily hold. This knowledge here rules in the bottle and rules out the table’s surface.

##### Laterally ambiguous gestures

In a comparable example, a gestural cue and a core cue again combine to provide complementary information about the target, thus enabling the hearer to single it out. Here ambiguity and its resolution are brought into the analysis. Suppose that a speaker in conversation with a companion wants to target a woman standing together with a man across a room with other women and men. The speaker points toward the couple while saying (5a):

(5) a. She is the director of our lab.
b. That is the director of our lab.

The gesture rules in the couple while ruling out the other people in the room, but the speaker is far enough away that it cannot indicate which member of the couple is intended. We will say that this gesture has *lateral ambiguity*. The trigger *she* provides the core cues that the target is uniplex, an entity, animate, female, and third-person. This cue then provides the additional information needed for the hearer to narrow down to the woman of the couple as the intended target. We will say that the core cue here enables the *lateral disambiguation* of the gestural cue. Note that if the speaker had said (5b), the trigger *that*–indicating only that the target is uniplex, an entity, distal, and third-person–would not provide enough information for such lateral disambiguation.

The process of disambiguation here can also be regarded as proceeding in the reverse order. Thus, the trigger *she* in (5a) rules in all the third-person women in the room as candidates for target status while ruling out all the men (as well as other entities), but it does not narrow this selection down to the woman in the couple. The gestural cue then provides the additional information needed to zero in on that particular woman.

#### Corporal Cues

The sheer presence of the speaker’s–or, in certain cases, the hearer’s–body at a particular location in space at the time of the speaker’s utterance can serve as a *corporal cue* to the speaker’s intended target. If the hearer is not already aware of this location, he must determine it perceptually or establish a mental image of it (as in a phone conversation) in order to make use of it as a cue. A corporal cue does not need to be accompanied by a gestural cue.

To illustrate, a woman in a booth at a fairground could reply as in (6) when asked by someone standing in front of her about the whereabouts of a certain man, Fred.

(6) Fred was here earlier.

In her utterance, the trigger *here* has its “corporal reading” (some languages have a distinct morpheme for this sense). Specifically, in addition to directing the hearer to find a target, it provides the core cue that that target is the spatial region surrounding the speaker’s current location. The hearer combines this core cue with the corporal cue consisting of the speaker’s actual location, which he perceives directly (or would imagine if on the phone with her). This cue combination allows him to select the region immediately around the woman out of all possible regions as the target. The speaker did not need to gesture–for example, by pointing to the ground in front of herself–but relied on the hearer’s determining her bodily location. The utterance goes on to indicate that the man asked about was previously situated in the region now being characterized.

### The Collateral Cue Categories

When a speaker initiates a targeting event, the entities that she talks about and to–namely, the target and the hearer–can be regarded as categories generated by and collateral to the speaker. Cues to the target provided by these two collateral entities, then, belong to a group of two *collateral cue categories*. Cues provided specifically by the target and by the hearer are treated next in order.

#### Targetive Cues

A speaker’s target generally exhibits characteristics that the hearer can discern, whether immediately or after a search. These characteristics can serve as cues for the hearer. A speech-external target in particular can produce sensory stimuli that provide the hearer with perceptual cues. These cues help guide the hearer toward the very entity producing them, which he can then single out as the target. These cues that the target itself provides will be called *targetive cues*. Two main types of such cues, the feature type and the salience type, are discussed next in order.

##### Targetive Feature Cues

Any intrinsic or contingent feature that a target exhibits, such as its own identity or its current distance away, can serve as a *targetive feature cue*. Its use was already seen in the “she is new” example in (1a). The trigger *she* in the speaker’s utterance there provided the hearer with the core cues that the target had the features of being uniplex, an entity, animate, female, and third-person. In processing the visual scene before him, the hearer perceived one element with those same features–the woman in the lab. Taking these latter features as targetive feature cues then allowed him to combine them with the core cues–they corroborated each other–and to settle on that entity as the intended target.

A related example rests on a co-form cue instead of core cues. As they round his house onto an open field with a tractor, a horse, and a car spaced apart in the distance, a farmer says (7) to a visitor without gesturing or looking at the tractor:

(7) That is my tractor.

In addition to the usual core cues from the trigger *that* here, the co-form cue from the noun *tractor* ascribes to the target the feature of being a tractor in its identity. The physical tractor in the field, in turn, provides the visual stimulus of being a tractor in its identity. This is then the targetive feature cue. The two cues corroborate each other and help the hearer zero in on the tractor in the field–not, say, on the horse or the car–as the intended target.

##### Targetive Salience Cues

Where the features that a speaker’s utterance ascribes to the target are insufficient for its determination, the hearer can instead search his perceptual environment for the most salient phenomenon within it, as judged on the basis of some 20 salience-associated parameters (Talmy, 2018; section 7.2.2). He entertains this phenomenon as a target candidate that exhibits *targetive salience cues*.

To illustrate, an experienced camper at a lake with a novice companion might, without gesturing, say (8) just after what seemed like a long plaintive sound could be heard:

(8) That is a loon.

The trigger *that* in the speaker’s utterance directs the hearer to search for a target and provides the core cues that it is a uniplex distal third-person entity. The noun *loon* provides few co-form cues to the hearer, who is less familiar with the word. These cues together do not ascribe enough features to the target for the hearer to determine it. He instead performs a salience search of his environment.

The sound that the hearer has just heard has several forms of salience. It is unique in its surroundings, non-prototypical for its category, and unfamiliar to the hearer. This salience of the sound constitutes a targetive salience cue. This cue then tends to rule that sound in as a target candidate and to rule out other concurrent sounds or, for that matter, non-sonic phenomena without salience. Furthermore, the temporal nearness of the sound to the trigger’s moment of occurrence provides a perichronal cue (see below) that tends to rule the sound in as a target candidate while ruling earlier-occurring sounds out. Combining these cues, the hearer is likely to settle on the long plaintive sound as the speaker’s intended target^6^.

#### Hearer-Focus Cues

A *hearer-focus cue* is a cue, metacognitively available to a hearer, indicating that her own current object of attention may be the speaker’s intended target. In that case, she must also be sure that both the object and her attention on it are perceived by the speaker. To illustrate, a speaker who sees his friend looking fixedly at one particular car among others on the road might say (9) to her without himself gesturing:

(9) That is a Ferrari.

The trigger *that* in his utterance directs the hearer, his friend, to ascertain any available cues and use them to determine a target that he has in mind. The trigger itself provides her with the core cues that the target is a uniplex, distal, third-person entity. However, she does not find a gestural cue or a targetive cue from some especially salient object in the environment to help with her search. Among the additional cues she can check for is her own current focus of attention. She metacognitively notes that the object of her attentional focus is the car she is gazing at, and she is aware that the speaker can see both the car and her fixed look at it. In the absence of more compelling cues, she accepts the direction of her attention as a hearer-focus cue. She combines it with the core cues to settle on the car she is looking at as the target that the speaker aims to communicate about with his utterance.

### The Background Cue Categories

In a still further group of two categories, the *background cue categories*, cues to the target arise from an extended field of phenomena–from the surrounding environment in one category and from the hearer’s own cognitive infrastructure in the other.

#### Environmental Cues

The “environment” is everything that extends out from the speaker in the speech-external domain and from the trigger in the speech-internal domain. An *environmental cue* then is any information provided by a component of the environment that helps the hearer determine the target. In the two domains, respectively, such information consists of physical stimuli that the hearer can perceive and of syntactic properties that the hearer can discern. Environmental cues are here chiefly divided into ones that help a hearer either locate a target or bound it, addressed next in order. A secondary division that crosscuts the first rests on whether an environmental cue involves content or structure.

##### Environmental locating cues

*Environmental locating cues* come from aspects of content and structure within the total environment that guide the hearer in narrowing down to just a certain subenvironment that the target is located in. This reduction process thus limits the search space that the hearer must check through to find the target. We here sketch two variants of such reduction. As one of the variants, the subenvironment can be a continuous region that encompasses the target. To illustrate, a speaker on a farm might, without gesturing, say either (10a) or (10b) to a visitor:

(10) a. That Cessna in the field is Jane’s.
b. That Cessna in the air is Jane’s.

The core cue from the trigger *that* and the co-form cue from the noun *Cessna* direct the hearer to search for a uniplex, distal, third-person entity with the identity of a Cessna. The prepositional phrase in each utterance provides further co-form cues that help the hearer limit her search. Both phrases direct the hearer to attend perceptually to the surrounding environment and to abstract out certain aspects of its structure and content. For both phrases, in fact, this abstraction here includes the horizontal layer of space directly above and adjacent to the horizontal plane of the land. More specifically, the hearer knows from the phrase in (10a) that she can limit her search to the horizontal layer of space at her own eye level just above the land and can dispense with looking up or down. She knows from the phrase in (10b) that she can limit her search to the space overhead and omit looking through the space at eye level or below.

In the second variant, the subenvironment is not continuous but consists of a set of distinct elements, one of which will be the target. To illustrate, a speaker might, without gesturing manually or looking, say (11) to a hearer as they stand in a field with a number of cows and horses, where one of the latter is gray:

(11) That gray horse is Jane’s.

The co-form cue from the noun *horse* may first lead the hearer to reduce her attention down to all the elements of the environment with the identity of a horse–together constituting the subenvironment–thus excluding the cows and other entities. Guided by another co-form cue from the adjective *gray*, the hearer needs then only look through that subenvironment–that is, through the set of horses–to find the gray one as the target. This succession of reductions constitutes a *nested search*. The hearer need not search directly through the entire environment for all occurrences of a gray color.

##### Environmental bounding cues

*Environmental bounding cues* are aspects of content and especially structure in the environment that help a hearer determine the outer boundary of what the speaker intends as his target. The hearer is generally guided to a particular set of such environmental aspects by cues of other categories.

To illustrate, as they stand atop a hill near a lagoon, a speaker might say (12) to a hearer while pointing at the middle of the lagoon:

(12) !-Mist forms there at night.

The trigger *there* in the speaker’s utterance initiates the hearer’s targeting procedure and provides the core cue that the target is a distal location. At the same time, the speaker’s pointing gesture may lead the hearer to imagine an intangible line extending from the finger to one point at the lagoon’s center. The hearer interprets this as a gestural cue to the target, but is that target to be the one point or some larger area around it? The hearer’s general knowledge provides the epistemic cue (see next) that mist does not form at a single point but over some area; but then, what area? An environmental bounding cue provides this final information about the target. The hearer perceives that an area of roughly uniform appearance extends from the gesturally indicated point out to the lagoon’s perimeter. This perimeter is a structural delineation within the environment. The hearer thus settles on the target as being not the spot pointed at but the entire surface of the lagoon as bounded by its outer perimeter.

#### Epistemic Cues

An *epistemic cue* is any information that a hearer derives from his own knowledge and beliefs that then helps him determine the speaker’s intended target. Two main types of this cue category are knowledge about entities and knowledge about discourse, illustrated next in order.

##### Epistemic entity cues

To illustrate entity knowledge, after they get off a train, a speaker might say (13) to a companion beside her while pointing toward three people–two men and a woman–waiting for her in the station. One of the men looks substantially older and the other younger than the speaker.

(13) That is my father.

As before, the trigger *that* in the speaker’s utterance initiates a targeting procedure in the hearer and provides the core cue that the target is a uniplex, distal, third-person entity. By itself, this cue does not much reduce the set of target candidates since there are many such entities in the scene. The gesture does narrow this set down to the three people in its scope. Since its distance away gives rise to lateral ambiguity, it does not indicate which of these three is the intended target.

In addition, though, the word *father* provides the co-form cue that the target is a man who has sired a child. The phrase *my father* provides the further co-form cue that the targeted man has sired the speaker herself. The “man” component within the semantics of these co-form cues provides further complementary information that rules out the woman and narrows the target pool down to the two men of the trio, but neither of these co-form cues by itself distinguishes between the two men.

However, the word *father* also activates the conceptual category “father” in the hearer’s knowledge store, which, besides other information, provides the epistemic cue that a father is older by some years than his child. The combination of this epistemic cue with the phrasal co-form cue indicates specifically that the targeted man is older than the speaker. The further combination of this result with the environmental content cues provided by the two men’s appearances finally leads the hearer to rule out the younger-looking man and to conclude that the target is the man in the pair of men who looks older than the speaker.

##### Epistemic discourse cues

To illustrate discourse knowledge, two zoo visitors are in front of an enclosure with a giraffe and a straight-horned antelope standing close together, and one says (14) while pointing with lateral ambiguity at the pair of animals:

(14) That is an oryx.

In the hearer’s knowledge of discourse management, a speaker would not state as new information something that the hearer would be expected to already know–here what a giraffe and the word for it are. Using this as an epistemic cue for lateral disambiguation, she concludes from the speaker’s use of the unusual word *oryx* that the trigger *that* targets the animal in the pair other than the giraffe.

### The Temporal Cue Categories

In this final group of two *temporal cue categories*, cues to the target arise from the temporal characteristics of elements present in an event of targeting. Cues of this sort from the trigger are in one category and cues from non-trigger elements are in the other.

#### Chronal Cues

The sheer occurrence of a trigger in a speaker’s utterance at a particular location in time can serve as a *chronal cue* to the speaker’s intended target. For certain triggers, such as English *now*, this target is itself an interval of time, one that extends through the moment of that trigger’s occurrence. The speaker’s utterance, furthermore, regularly identifies a particular state or event that occurs within or throughout this targeted interval. We can illustrate with a speaker saying (15) to a guest in her house:

(15) The bathroom is free now.

The trigger *now* in her utterance directs the hearer to determine the target, and it provides the core cue that this target is a temporal interval. It further indicates that this interval extends through her trigger’s moment of occurrence. This latter indication, in turn, rests on what can be analyzed as an independent process, namely, determining that trigger’s moment of occurrence. That is, the hearer must determine the chronal cue–the moment at which the speaker’s trigger is uttered–and combine it with the core cue so as to center the interval around that trigger moment. As it happens, determining this chronal cue is straightforward, consisting simply of the hearer’s taking cognizance of the moment at which he just heard the trigger.

In addition, the hearer’s knowledge about bathroom use provides the epistemic cue that the length of the targeted interval should be reckoned in minutes–rather than, say, hours or, for that matter, decades, as would be the case for the interval targeted by the *now* in the sentence *We are in the age of the Internet now*^7^.

The hearer concludes that the target is an interval of some minutes passing through and centered on the trigger he has just heard. He then temporally locates the state referred to by the utterance–the bathroom’s availability–as occurring throughout this interval.

#### Perichronal Cues

A *perichronal cue* is any temporal property of an element other than the trigger that helps the hearer determine that trigger’s target. In the majority case, though, perichronal cues do not help determine the target directly. They are rather the temporal properties of elements near a trigger that help determine which of those elements can serve as cues to its target, ruling some of them in and others out on the basis of their timing.

To illustrate, suppose that two joggers are running along the sidewalk past successively parked cars spaced amply apart, each car in turn to the left of them. At one point, one runner says (16a) to her companion while pointing leftward and, a few moments later, says (16b) while again pointing leftward:

(16) a. That is my car.
b. And that is my sister’s car.

To examine the second communication, the trigger *that* in the speaker’s (16b) utterance directs the hearer to find a particular target and provides the core cues that it is a uniplex, distal, third-person entity. In addition, the co-form cue from the word *car* tells him to look for a car as that target. Furthermore, the gestural cue from the speaker’s second pointing movement and the targetive cue from the car appearing directly in view on the left both provide the perichronal cues that their occurrence is close enough in time to that of the trigger for them to be relevant.

By contrast, the previous (16a) pointing movement and the car it pointed at are ruled out as providing gestural and targetive cues relevant to the present (16b) trigger. Though the hearer might, in principle, entertain them as potential cues, their time of occurrence is too distant from that of the current trigger, so he concludes not to use them as indications of the speaker’s currently intended target. Thus, through his observation of perichronal cues, the hearer takes into account only the concurrent gesture and the car immediately to his left as pertaining to the present event of targeting and disregards the earlier gesture and other cars along the curb.

## Interaction of Compatible Cues to a Speech-External Target

While the preceding survey focused on one cue category at a time, each example in the following sections includes numerous cues of different categories to allow attention to their interactions as well as to the hearer’s processing of them.

Our account of this processing, it must be emphasized, is in the form of a *regularized description*–consisting of a succession of discrete steps–both for clarity of presentation and to reflect logical relations. Such a description is not based on assumptions about any actual operations in cognitive processing, which may well occur in parallel or in other sequences as well as being more gradient than discrete and which must be determined experimentally. We do posit, though, that the elements and the relationships presented in the description are *somehow* represented in cognition since any absence among them would disrupt targeting. The detailedness of the description, furthermore, may help identify the numerous potential points of articulation in cognition relevant to such processing. Their presence in cognition is indeed posited on the assumption that little in cognition just happens by itself, no matter how quickly and unconsciously a hearer’s processing may proceed or how self-evident the result may seem.

The examples in the present and the next section have only compatible cues. The cues in these two examples are, respectively, to a speech-external and to a speech-internal target, but the example in section “Interaction of Incompatible Cues to a Speech-External Target Broader Systems” includes conflicting cues.

In the present illustration involving a speech-external target, a couple walking along a sidewalk stop a foot in front of a gift shop window and look in. The speaker knows that her companion had wanted to learn what the color puce looks like among other colors that he was unclear about, such as vermilion and chartreuse. The speaker spots puce coloring among the gift items on display and–wanting to direct the hearer’s attention to it–says (17) while facing toward the interior and pointing:

(17) Those boxes are puce-colored.

In the display’s setup, a platform extends back behind the shop window. A single cluster of gift items appears in the front portion of the platform, while three separate clusters are arrayed left to right along the rear portion. The middle cluster in the rear includes some boxes that are red, some boxes that are of a hue which is unknown to the hearer–hue number 1, a single box with unknown hue number 2, and some statuettes of unknown hue number 3. The front cluster has some boxes of unknown hue number 4. While saying (17), the speaker points toward the rear middle cluster, but her gesture is laterally ambiguous, unable to single out specifically what within the cluster is puce-colored.

The trigger *those* in the speaker’s utterance sets the hearer off on a three-stage targeting procedure. In addition to other information, the trigger provides the core cue that the target is distal. This core cue rules out any proximal entities from being the target. However, the hearer cannot use this cue by itself because the distal/proximal distinction that it entails is topological. It could distinguish between entities separated by inches as readily as by miles. It must be combined with information from another cue, an operation described below.

At the same time, the orientation of the speaker’s head and body provides a gestural cue, namely, that the target is situated within the corridor of space extending forward from her front. This gestural cue thus rules out all entities located outside this corridor as candidates for target status.

The platform that the hearer perceives nearby within this corridor of space can then provisionally suggest an environmental locating cue. This cue is that the perimeter defining the platform’s expanse is also the boundary of the region in which the target is located. If confirmed, this cue would then eliminate any other regions in the corridor as areas in which the target might be found.

The hearer can now combine this environmental locating cue with the earlier-mentioned core cue that the target is distal. Now anchored on the platform, that cue loses its topological relativity and rules in the rear portion of the platform while ruling out the front portion. This fact also eliminates the possibility that the target is in the cluster of gift items located in that front portion. Accordingly, puce cannot be the unknown hue 4 of the boxes in that cluster.

A second gestural cue–the one provided by the speaker’s pointing finger–is not precise enough to pinpoint the target, but it is precise enough for certain other indications. First, it corroborates the environmental locating cue that the target is situated within the perimeter of the platform, thus confirming a cue that, in this regularized description, was previously only provisional. Second, it corroborates the just-seen indication that the target is located in the rear of the platform. This indication itself had been derived by combining the environmental locating cue with the core cue. Third, it provides the new indication that the target is located in the region of the middle cluster out of the three clusters along the platform’s rear. This gestural cue thus rules out both side clusters from consideration.

In addition to its core cue that the target is distal, the trigger *those* provides the core cue that the target has an “entity” character. This cue thus rules out the possibility that the target is a location, among other non-entity-like options. If it had been viable, such a location, in accordance with the preceding cues, could have been the volume of space occupied by the middle cluster or the portion of surface it rests on. What this additional core cue does rule in as target candidates, then, is either a particular physical object or objects within the cluster or the full ensemble of the cluster.

A third core cue provided by the trigger *those* is that the target is third-person, which excludes the speaker and the hearer as target candidates. This core cue thus corroborates the same exclusion indicated by the two gestural cues, which located the target where the speaker pointed at within the corridor defined by her bodily orientation.

Additional information next comes from the morpheme *box* that the speaker uses in her utterance. It provides the co-form cue that the target has a “box” identity. At the same time, through the visual stimuli they produce, the statuettes in the middle cluster provide the environmental content cue that they have a “statuette” identity, while the remaining items in that cluster provide the environmental content cue and potentially targetive cue of having a “box” identity. In conjunction with these additional cues, then, the co-form cue rules out the statuettes in the cluster as candidates for target status–thus eliminating unknown hue number 3 as puce–but rules in the remaining items. This co-form cue also corroborates the preceding two core cues in the elimination of the collocutors and of any locations from target candidacy.

An additional co-form cue comes from the plural morpheme *-es* on *box*, which indicates that the target is multiplex. At the same time, the trigger *those*–in addition to its core cues that the target is distal, entity-like, and third-person–provides the core cue that the target is multiplex. This final core cue and the co-form cue from the plural morpheme *-es* thus corroborate each other in their indication that the target is multiplex. This redoubled indication thus rules out the single box of unknown hue number 2 as a candidate for the target, but they still leave the red boxes and the boxes of unknown hue number 1 as candidates.

What then distinguishes between these two candidates is an epistemic cue from the hearer’s knowledge of discourse principles. He knows that the speaker, following an informativeness principle, would not present as new information something that her addressee would be assumed to know already, such as what red is. On the basis of this epistemic cue, the hearer reasons that the speaker could not have been informing him about the red boxes and hence must have been referring to the last remaining target candidate, the boxes of unknown hue number 1.

The hearer still has more cues to note and narrowing down to do. The present tense of the verb *are* can be regarded as a second trigger in the speaker’s sentence. It provides the chronal cue that the target occupies its location during an interval that extends through the moment of the trigger’s utterance–that is, the current moment. The target thus does not occupy its location during an interval wholly before or after this current moment. This cue thus eliminates from potential target status any puce-colored boxes that may have been present in the past or might be present in the future at the indicated location. If the speaker had intended such a target in, say, the past, she would instead have said something like (18a or b). With such temporally displaced boxes ruled out, the presently appearing boxes of unknown hue number 1 continue to be ruled in.

(18) a. Those boxes were puce-colored (yesterday).
b. Some boxes there (yesterday) were puce-colored.

Finally, suppose that the couple had also been stopping at shop windows at other locations along the street, pointing at and commenting on items in them. Some factor in the hearer’s cognition must be present that leads him to deal only with the gestural and the targetive cues concurrent with the present trigger and associate these all with each other rather than to use cues from the recent past now in memory. The concurrentness of the present gestural and targetive cues is a perichronal cue that rules them in, while the non-concurrentness of the previous cues is a perichronal cue that rules them out.

In sum so far, the hearer is prompted into a targeting procedure by the trigger *those* in the speaker’s utterance. In the first stage of this procedure, he discerns some dozen-specific cues from eight different categories. In the second stage, he integrates these cues to the point where they enable him to narrow down to the speech-external target evidently intended by the speaker. This target turns out to be the boxes of unknown hue number 1. The narrowing-down process has ruled out any other items currently visible in the window display, any items that were or will be in that display, any items that were or will be seen in other displays along the way, and, generally, any non-items or items outside the display.

In the third stage of the procedure, the hearer next maps the concept of the now-identified target back onto the trigger *those*. In accord with the trigger’s syntactic relation to the sentence–namely, as the determiner of a subject nominal within a construction of predicate–adjective attribution–he integrates that concept into the sentence’s overall conception. As a final result, he concludes that the boxes he has perceptually narrowed down to, tinted with one of the hues he had not known, are in fact puce-colored.

## Interaction of Compatible Cues to a Speech-Internal Target

Shifting focus from a speech-external to a speech-internal target, we provide an illustration in which a man and a woman are alone in a room, and he says the two consecutive sentences in (19) to her.

(19) a. My sister led her mare down the hill toward some cowboys.
b. She was dappled.

The trigger *she* in the speaker’s (19b) utterance directs the hearer to undertake a three-stage targeting procedure. It also provides the core cues that the target is uniplex, an entity, animate, female, and third-person. Although the hearer herself has the first four of these characteristics, she lacks the fifth. Relative to the speaker, the hearer is not third-personal but second-personal and, if the speaker had intended her as the target, he would have instead used the trigger *you*. Accordingly, the core cue that the target is third-person rules out the hearer herself as a possible target but rules in other female animate entities as candidates for target status.

The hearer might accordingly look for the speaker’s intended target in her physical surroundings. From her perception of those surroundings, environmental content cues arise with the information that no such female beings are present there. These environmental cues thus suggest ruling out the speech-external environment as the target’s location.

This suggested exclusion may then be corroborated in the hearer’s cognitive processing by the state of the cues from three further categories. First, there is an absence of gestural cues–the speaker does not produce targeting gestures as he speaks. This suggests that, in the immediate physical surroundings, the target is not present to be gestured at. Second, in full corroboration with the environmental content cues, there is an absence of targetive cues provided by perceptual stimuli from another woman. This again suggests that the target is not present in the immediate physical surroundings. Third, the tense of the verb *was* in (19b)–which can be regarded as a second trigger in that sentence–provides the core and the chronal cues that the target’s time of occurrence is not that of the trigger itself, that is, the present. If it had been, it too might have indicated a target in the current immediate physical surroundings. These multiple indications to rule out the speech-external environment as the target’s location then strongly suggest ruling in the speech-internal environment as its location.

To find the target, the hearer thus directs her attention to the speech-internal environment. This consists of the discourse–both its formal and its semantic aspects–that the trigger *she* occurs in. The perichronal cues from this discourse increasingly rule out portions of it the further they are in time from the trigger’s occurrence and increasingly rule in portions of it the closer they are. These perichronal cues may finally narrow the location of the target down to the utterances in (19) themselves and eliminate utterances outside them.

The hearer may next consider environmental content and structure cues present in the discourse surrounding the trigger (rather than considering them in her physical surroundings, as she had done earlier). These cues consist of information about the formal and the semantic components of the utterances that have been ruled in through perichronal cues and indicate that the target is likely to be one of those components.

To consider the formal components of an utterance first, they generally consist of its morpheme, word, and phrase constituents as well as the grammatical relationships that these bear to each other. The formal components of (19a) include four noun phrases, a verb, and two prepositions as well as their contained and containing constituents and all the grammatical interrelationships present. Those in (19b) include a trigger, a verb, and an adjective as well as their grammatical relationships.

For their part, the semantic components of an utterance generally consist of the meanings of the formal components and their relationships as well as of their pragmatic implications. The semantic components of (19a) include the speaker’s sister, her mare, a hill, some cowboys, an act of leading, and a path of descent and approach, among other indications and relationships. The semantic components of (19b) include a quality of dappledness and, from the trigger *she*, a directive to find a target together with core cues to that target.

These core cues can now interact with the remaining environmental content cues. A first result comes from the core cue that the target is animate. This cue actually eliminates all the formal components of both utterances from the possibility that the target is one of them. The reason is simply that formal linguistic components are never animate. This elimination leaves only the semantic components within the two utterances as candidates for target status.

But then, this same core cue that the target is animate further eliminates some of these semantic components as well. To begin with, it eliminates all the semantic components of (19b) since none of them–e.g., neither the dappledness nor the trigger’s directive or core cues–is animate. This core cue eliminates itself as well because it is, in fact, not itself an animate but rather part of a directive to find an animate.

These exclusions then leave only the semantic components of (19a) as contenders for target status. Now another core cue provided by the trigger *she*–that the target has an “entity” character–rules out such semantic components as the act of leading and the downward approaching path. It rules in only four semantic components: the speaker’s sister, her mare, a hill, and the cowboys.

With respect to these four ruled-in components, the core cue that the target is animate again comes into play to rule out the hill. At the same time, the core cues that the target is uniplex and female both rule out the cowboys. Among the semantic components of the first utterance, then, the core cues together rule in the speaker’s sister and her mare as candidates for target status but rule out the rest.

The hearer’s ability to select a single target from these two remaining candidates is furthered by information from the co-form *dappled*. This adjective provides the co-form cue that the target has the property of being “dappled”. This adjective has two main meanings: one involves spots of different shades intrinsically present on the skin or fur of a non-human animal; the other involves spots of light being reflected off of any surface. If the first meaning is in effect, the co-form cue from the adjective is enough to finally narrow the selection down to the mare because the mare is a non-human animal while the sister is human; but if the second meaning is in effect, the co-form cue does not distinguish between the two remaining candidates since they both present surfaces.

An epistemic cue then finally enables the hearer to zero in on the target. This cue is the hearer’s linguistic knowledge that, for the second meaning of the adjective *dappled* to be in effect, the adjective must be accompanied by a phrase referring to light or shade. This meaning would be evoked, for example, in a sentence like that in (20).

(20) My sister was dappled in the sunlight (that filtered through the leaves of the trees).

However, the utterance in (19b) did not include such a phrase. Accordingly, the hearer concludes that only the first meaning of *dappled* can be in effect. Thus, the hearer finally settles on the mare as the speaker’s intended target.

In sum to this point, the trigger *she* in the speaker’s second utterance initiates a targeting procedure in the hearer. In the first stage of the procedure, the hearer discerns over a dozen either negative or positive cues–that is, cues that are missing or that are present with specific content–from seven different categories. In the second stage, her processing of these cues enables her to narrow down to the speech-internal target intended by the speaker. This target turns out to be the mare referred to in the speaker’s first utterance. This narrowing-down process has ruled out everything in the current speech-external environment and, within the speech-internal environment, all the formal components and all but one of the semantic components.

In the third stage of the procedure, the hearer next maps the concept of the now-identified target back onto the trigger *she*. Since, as a semantic component, the target “mare” is already a concept, the hearer simply maps this concept–or a copy of it–onto the trigger. In accord with the trigger’s syntactic relation to the sentence–namely, as a subject nominal within a construction of predicate-adjective attribution–she integrates that concept into the sentence’s overall conception. She concludes that the mare in the discourse just referred to by the speaker has naturally dappled skin.

## Interaction of Incompatible Cues to a Speech-External Target

In the preceding two illustrations, the cues were all compatible, but here some are incompatible, providing conflicting information about the target (itself again speech-external). This incompatibility is a well-formed feature of the speaker’s production, designed to initiate cognitive processing in the hearer that resolves the conflict so he can proceed to determine the target.

In our illustration–sketched here but detailed in the book–a woman sits across a restaurant table from a man. For two initial control examples with unconflicted cues, she either says (21a) and points to the right side of her own mouth or says (21b) and looks at his mouth while extending her finger across the table to point directly at it:

(21) a. I have got something in my teeth right here.
b. You have got something in your teeth right there.

In the conflicted example in (22)–which has the same import as (21b)–she again looks at his mouth but, as in (21a), points to a spot on the right side of her own teeth.

(22) You have got something in your teeth right here.

In (22), the trigger *here* directs the hearer to determine the speaker’s intended target and, to that end, to determine the available cues to it, including the following five. The trigger itself provides the core cue that the target is a location proximal to the speaker. An environmental locating cue from the perceivable surroundings indicates that the speaker’s body is the setting for this targeted location. One of the speaker’s two gestural cues, the manual one from her finger, indicates that the targeted location is a spot on the right side of her teeth. Another gestural cue, the ocular one from her gaze, indicates that the targeted location is at the hearer’s mouth. The phrase *in your teeth* provides the co-form cue that the targeted location is at the hearer’s teeth.

The hearer’s processing of these cues he has assembled–again using a regularized description–begins with an assessment phase. Within this phase, an initial operation of *consistency checking* examines the cues for their mutual compatibility. It proceeds on the basis of a certain set of principles, including one of plausibility. Here taking all the cues at face value can lead to an implausible conception, such as one in which some of the hearer’s teeth are in the speaker’s mouth. He thus concludes that some of the cues in fact are in conflict.

A second operation of *clustering* within the assessment phase segregates the cues into groups that are each internally compatible but that are incompatible with each other. Here the co-form cue and the ocular gestural cue are compatible with each other in one group, both indicating that the targeted location is at the hearer. Incompatible with this first group is a second group that includes the core cue, the manual gestural cue, and the environmental cue–all compatibly indicating that the targeted location is at the speaker.

A third operation of *evaluation* within the assessment phase assigns opposite states of validity to the two incompatible groups mainly on the basis of a *problem-avoidance principle*. Control example (21a) lacks problems, but control example (21b) has some: her reaching gesture might be considered as socially inappropriate, physically awkward, or incapable of precision. By contrast, the conflicted example in (22), in which the speaker touches her own teeth, avoids such problems: it evades social stigma, is easier to perform, and permits precision (the hearer can now use his vision to learn the exact location).

The hearer concludes that the speaker has resorted to the conflicted utterance to avoid the problems of the direct communication in (21b) and hence that its import equates to that communication rather than to the unproblematic direct one in (21a). By the evaluation operation, then, the group of two cues that the conflicted communication shares with the direct one in (21b)–the co-form and ocular gestural cues–is assessed as *valid*, while the other group of three cues is assessed as *anomalous*.

The hearer’s processing next proceeds to a resolution phase. This phase retains the valid cues, that is, the co-form cue and the ocular gestural cue; but it adjusts the anomalous cues–that is, the core, environmental, and manual gestural cues–so that they become compatible with the valid cues. The full coherent set of cues that results can then lead to the intended target.

The main operation in this resolution phase is that of *mapping*. This operation acts on the location at the teeth in the speaker’s body that is seemingly targeted by the anomalous cues–the *initial target*. Through this mapping, the hearer imaginally translates that location to the structurally homologous location on his own body–the *final target*. *Rotational* mapping targets a location on the right side of his teeth, while *reflective* mapping targets a location on the left side.

## Broader Systems

We conclude by observing that the targeting phenomena presented in this overview are generally part of broader systems significant in their own right. We here cite some of these, all examined in Talmy (2018), as noted below.

First, the joint attention on a target that a speaker aims to achieve with a hearer is part of a broader system of *common attention* (Section 13.3, “Taxonomy of Common Attention”). Common attention is characterized by different combinations of settings along four parameters: participation, recognition, elicitation, and epistemic parameters. Although “joint attention” seems to be the prototype in communication and is the term usually found in the literature, in our analysis, it is not an elementary phenomenon but the most elaborated endpoint in a hierarchy of attentional patterns. It is a conjunction of the highest settings on all four parameters: elicited mutually recognized common attention based on observation.

Next, the pointing gesture of earlier illustrations is merely the prototype within a prodigiously extended system of targeting gestures (Chapter 5). In this system, the fictive chain imaginally generated by a gesture can alternatively intersect with, enclose, pervade, coprogress with, parallel, access, “behold,” neighbor, or contact a target at different levels of precision.

Furthermore, the system of fictive chains is part of a more general cognitive system of *spatial fictivity* (Section 5.15, “A Cognitive System of Spatial Fictivity”). This system is often engaged within visual perception, linguistic representation, and cultural constructs.

In addition, the phenomenon of conflict and its resolution was seen engaged with respect to cues to a target in section “Interaction of Incompatible Cues to a Speech-External Target Broader Systems” above. It is part of a much broader cognitive system. When at work in language, this system enlists *constructive discrepancy* and underlies all tropes, including that of metaphor (Section 14.4.1, “Conflict and Resolution in Tropes”).

Targeting can also be, in part, incorporated into other preexisting theories, but then it requires their expansion (Section 1.8.2, Specific Approach Comparisons). For example, triggers, as a linguistic category, constitute a certain type of construction within the broader theory of construction grammar, as articulated, for example, by Fillmore et al. (1988) and Goldberg (1995, 2006); but the trigger construction has novel properties whose inclusion necessitates the theory’s extension or again, indexicality theory as articulated, e.g., by Silverstein (1976, 2003) includes an index and its object, corresponding to our trigger and target. Where their index simply “stands for,” “represents,” “points to,” or “indexes” its object, targeting inserts an entire stratum between index and object, a stratum consisting of cognitive processes that ascertain cues and integrate them so as to narrow down to that object/target.

It can thus be seen that, through its own properties and their generalizations like those above, targeting is a significant area for exploration within cognitive science because of the window it opens onto the nature of cognition overall.

Note: All the author’s publications except the 2018 book are available on his website: https://www.acsu.buffalo.edu/t̃almy/talmy.html.

## Data Availability Statement

All datasets generated for this study are included in the article/supplementary material.

## Author Contributions

The author confirms being the sole contributor of this work and has approved it for publication.

## Conflict of Interest

The authors declare that the research was conducted in the absence of any commercial or financial relationships that could be construed as a potential conflict of interest.

## Footnotes

1 In another system of terminology, one not prominent in the United States, “anaphora” and “deixis” refer instead to an attentional timeline, distinguishing, respectively, between whether the hearer’s attention is already on the target when the speaker refers to it or is first brought to it by the speaker’s utterance. Talmy (2018) extensively analyzes this attentional distinction and the properties of the joint attention that either precedes or ensues but does so using terms other than “anaphora” and “deixis.”

2 To ensure such *cue adequacy*, the speaker cannot be heedless as to which cues may happen to be available but must function as a proactive agent so as to enable the hearer to determine the target. Any cue inadequacy, as with communication deficiency generally, can be repaired, e.g., if the hearer indicates the need for more information and the speaker then supplies it.

3 As analyzed in Talmy (2018, Chapter 2), many triggers across languages indicate a target’s *degree of remove* from the speaker (for a speech-external target) or from the trigger (for a speech-internal target). Such remove is not solely spatial but can be divided into five types: spatial, temporal, personal, social, and experiential; and the experiential type can be further divided into four subtypes: memorial, attentional, recognitional, and affective/perspectival. The number of different degrees that triggers distinguish varies by language and type of remove. But for spatial, temporal, and certain subtypes of experiential remove, English triggers distinguish only two degrees: *proximal* and *distal*. English triggers providing a core cue that a target is at a proximal degree of spatial remove (such as the trigger of the present example) include *this*/*these* and *here*, while those for a distal degree include *that/those* and *there*. Such triggers, like closed-class forms generally [see Talmy (2000a), Chapter 1], express concepts whose character is “topological” rather than “Euclidean.” In particular here, the proximal or the distal degree of a target’s spatial remove is “magnitude-neutral.” The degree of remove becomes more “magnitude-specific” only to the extent that other targeting cues (including elements of the context) narrow down the full possibility range. For example, other cues may set the distance between the two targets in (ia) and in (ib), respectively, at meters and parsecs, but the use of the proximal triggers *this*/*here* and the distal triggers *that*/*there* is unaffected by that difference. Their proximal/distal distinction only indicates that their target is, in effect, on the speaker-side or the non-speaker-side of a conceptual partition imaginally located midway between the two targets. These triggers are also neutral to the magnitude of their targets, which here–whether entities or locations–ranges from centimeters to megameters.

(i) a. This ball/The ball here is bigger than that ball/the ball there.
b. This planet/The planet here is bigger than that planet/the planet there.

4 In some cases in some languages, the morphological form of the trigger itself helps determine its semantic relation and hence how to integrate its concept. For example, the English triggers *he*, *him*, and *his* in a portion of discourse might all target the same male entity but indicate that the concept of that entity is to function within that discourse as, say, an agent, patient, or possessor, respectively.

5 A speaker’s targeting gesture is always at a different spatial location than her target. The hearer must have a cognitive mechanism that associates the gesture with the target (unlike, say, a cat that at most just looks at the gesture). Talmy (2018), Chapter 5 proposes that the hearer forms this connection by generating a fictive chain: a succession of imaginal constructs–possibly from a relatively closed universal inventory–that are either schematic (largely geometric) structures or operations that affect such structures. Such a fictive chain may have three properties of a physical mechanical system: (a) It is fully connected without gaps; (b) It forms progressively from the gesture to the target, not in place all at once nor from target to gesture; (c) It is causal: the gesture gives rise to the first fictive construct, the first construct to the second, etc. In the present example, the pointing finger may be schematized as a straight line with a front point that coaxially emits a straight one-dimensional projection that progresses quickly through space to terminate at and intersect with the location on the table to mark it as the intended target.

6 The trigger *that* targets this sound, itself in a metonymic relation with the loon–a relation that would be literal if the speaker had said *That is a loon’s call*.

7 This trigger *now* also exhibits magnitude neutrality (see text Footnote 3)–but here with respect to the size of the temporal interval it targets.
